# Early *in vivo* target genes in human immune cells highlight vitamin D’s role in antioxidant defense

**DOI:** 10.3389/fimmu.2025.1559486

**Published:** 2025-07-15

**Authors:** Tanya Tripathi, Carsten Carlberg

**Affiliations:** ^1^ Institute of Animal Reproduction and Food Research, Polish Academy of Sciences, Olsztyn, Poland; ^2^ Institute of Biomedicine, School of Medicine, University of Eastern Finland, Kuopio, Finland

**Keywords:** vitamin D, transcriptome, PBMCs, vitamin D target genes, immune system, detoxification

## Abstract

**Introduction:**

Vitamin D plays a vital role in modulating innate and adaptive immunity. This study investigated the gene regulatory mechanisms underlying this modulation *in vivo*.

**Methods:**

We conducted a proof-of-principle intervention in which a participant received a bolus of vitamin D_3_ (80,000 IU) monthly for three months. Peripheral blood mononuclear cells (PBMCs) were collected immediately before and at 4, 24, and 48 hours post-supplementation for transcriptome-wide differential gene expression analysis.

**Results:**

We identified 570 genes significantly responsive to vitamin D_3_ (p < 0.05) at one or more timepoints. *In vitro* experiments using PBMCs of the 0-hour time point of the same individual validated 303 of these as targets of the vitamin D receptor ligand 1α,25-dihydroxyvitamin D_3_. Among these, 55 primary target genes exhibited significant changes as early as 4 hours post-supplementation, including genes like *SELENOS* (selenoprotein S), which plays a key role in the selenium micronutrient network. Moreover, genes such as *PRDX1* (peroxiredoxin 1), *TXNRD1* (thioredoxin reductase 1), and *SOD2* (superoxide dismutase 2), involved in antioxidant defense, were prominently regulated.

**Discussion:**

These findings highlight a potential early and primary role for vitamin D in regulating detoxification processes, suggesting its critical involvement in maintaining redox homeostasis in immune cells of healthy individuals.

## 1 Introduction

Vitamin D_3_ is a vital micronutrient that can either be synthesized endogenously in the skin upon exposure to UV-B radiation (1, 2) or obtained through dietary sources and supplementation (3). One of the most ancient evolutionary roles of vitamin D is maintaining energetic and survival homeostasis, such as detoxification (4, 5). However, its most well-known physiological function is regulating calcium homeostasis, which is crucial for bone mineralization (6). Beyond these roles, vitamin D also plays a critical part in modulating the immune system (7–12). It supports the innate immune response to infectious diseases, such as tuberculosis (13) and COVID-19 (coronavirus disease) (14), while also preventing overactivation of the adaptive immune system. This dual function is essential for reducing the risk of autoimmune diseases, such as multiple sclerosis (15, 16), and for mitigating severe immune responses, such as those observed in critical cases of COVID-19 (17).

The vitamin D_3_ metabolite 1,25-dihydroxyvitamin D_3_ (1,25(OH)_2_D_3_) binds to and activates the transcription factor and the (vitamin D receptor) (18–20), thereby exerting direct effects on gene regulation (21). VDR, a member of the nuclear receptor superfamily (22), regulates several hundred specific target genes across approximately half of human tissues and cell types (23, 24). As a result, the biological functions of vitamin D_3_ in health and disease are intrinsically linked to 1,25(OH)_2_D_3_-mediated changes in the transcriptome of VDR-expressing cells (23). The vitamin D-regulated transcriptome has been extensively studied using various *in vitro* cell culture models (9, 25–27), including THP-1 monocytic leukemia cells (28). However, primary cells, which more closely mimic the human *in vivo* context, offer a more physiologically relevant alternative. PBMCs are particularly attractive for such studies, as they can be obtained from donors with minimal invasiveness (29). PBMCs consist of a diverse population of innate and adaptive immune cells, including monocytes, natural killer cells, and T and B lymphocytes. Among these, monocytes are the most responsive to vitamin D (30). The genome-wide binding pattern of VDR has been characterized in various human *in vitro* cell culture systems (31). In monocytes, the VDR cistrome comprises over 10,000 loci, although only a few hundred persistent VDR binding sites are consistently occupied (32). These persistent sites serve as the primary genomic contact points for 1,25(OH)_2_D_3_, orchestrating its spatiotemporal response as a nuclear hormone. The chromatin model of vitamin D signaling (33, 34), derived primarily from studies in THP-1 cells stimulated for 24 hours with 1,25(OH)_2_D_3_, proposes that the regulation of a primary vitamin D target gene depends on the presence of a prominent VDR binding site within its TAD (topologically associated domain). Since TADs range in size from 100 kb to 2 Mb, this defines the maximum distance between a VDR-binding enhancer and the TSS (transcription start site) of its corresponding target gene (35).

This study investigates transcriptomic changes in PBMCs at 4, 24, and 48 hours following vitamin D_3_ supplementation in a healthy individual. These *in vivo* data were compared with previously published (36) *in vitro* data obtained from PBMCs of the same individual. The time-resolved analysis provides a comprehensive view of the dynamic effects of vitamin D on these immune cells and highlights the stimulation of detoxification processes as early response to the prohormone.

## 2 Materials and methods

### 2.1 Sample collection

Peripheral blood samples were obtained from a single healthy participant (male, age 57) enrolled in the VitDHiD trial (NCT03537027, ClinicalTrials.gov) (37), which had been classified as a high vitamin D responder. The vitamin D status, expressed by 25-hydroxyvitamin D_3_ (25(OH)D_3_) serum levels, as well as the vitamin D_3_ concentration was measured using UPLC (1290 Infinity II LC (liquid chromatography) System, Agilent) coupled with MS (mass spectroscopy) detection (API 4000 LC-MS/MS System, SCIEX). The study protocol was approved by the Ethics Committee of the Northern Savo Hospital District (Approval #515/2018). Written informed consent was obtained from the participant, and all experiments were conducted in compliance with applicable ethical guidelines and regulations.

### 2.2 PBMC isolation

Peripheral blood samples (8 ml) were collected immediately before (0 hours) and at 4, 24, and 48 hours following a vitamin D_3_ bolus supplementation (80,000 IU). PBMCs were isolated within one hour of collection using Vacutainer CPT Cell Preparation Tubes with sodium citrate (Becton Dickinson) according to the manufacturer’s protocol. After isolation, the cells were washed with phosphate-buffered saline, aliquoted at a concentration of 4 million cells per ml, and stored at -80°C for subsequent RNA isolation. This experiment was repeated across three consecutive months using the same individual. In a previously published study (36), PBMCs isolated from the same individual at the 0-hour timepoint of each of the three biological replicates of the *in vivo* experiment were stimulated *in vitro* with 1,25(OH)_2_D_3_ for 4, 24, and 48 hours. This paired design ensured that the starting conditions of the present *in vivo* study were directly aligned with those of the *in vitro* experiment, enabling meaningful comparison between the two settings.

### 2.3 Transcriptome analysis

Total RNA was extracted from PBMCs using the High Pure RNA Isolation Kit (Roche) according to the manufacturer’s instructions. RNA quality was assessed using the Agilent 2100 Bioanalyzer system, ensuring a RNA integrity number ≥ 8. Library preparation was performed after rRNA depletion using kits and protocols from New England Biolabs. RNA-seq libraries underwent quality control on the Agilent 2100 Bioanalyzer before sequencing on a NextSeq 500 system (Illumina) with a 75 bp read length, following standard protocols at the EMBL Gene Core facility in Heidelberg, Germany. All samples were processed and sequenced in a single batch. Fastq files for the 12 libraries have been deposited in the Gene Expression Omnibus (GEO) under accession number GSE283231 and the raw data of the *in vitro* samples are available under accession number GSE189984. Sequencing quality was evaluated using FastQC (version 0.12.1, www.bioinformatics.babraham.ac.uk/projects/fastqc), with results summarized in **Supplementary Table S1**. In parallel, raw data from a previously published time-course experiment using PBMCs from the same individual, stimulated *in vitro* with 10 nM (36) (OH)_2_D_3_ for 4, 24, and 48 hours (36), were reanalyzed using the latest version of the human genome. Reads were aligned to the GRCh38 reference genome (Ensembl version 111.38) using the STAR aligner (38) (version 2.7.10b), and quantification was performed with FeatureCounts (39) (version 2.16.0) using default parameters. To ensure consistency in gene nomenclature, Human Gene Nomenclature Committee (HGNC) symbols were updated using the R package *HGNChelper* (version 0.8.1, https://CRAN.R-project.org/package=HGNChelper). Annotation, including gene identifiers, descriptions, genomic locations, and biotypes, was integrated from the Ensembl database (release 109) using the R package *BiomaRt* (40) (version 2.58.2). Entrez Gene identifiers were added using the R package *org.Hs.eg.db* (version 3.18.0), and any incomplete mappings for target genes were manually verified and retrieved from NCBI (www.ncbi.nlm.nih.gov/home/genes). Genes without genomic position information or those encoded in mitochondrial DNA were excluded from further analysis.

### 2.4 Differential gene expression analysis

Differential gene expression analysis was conducted in R (version 4.3.1) on MacOS 13 (Ventura) using the *DESeq* package (version 4.0.16) for robust assessment. To reduce transcriptional noise associated with non-coding genes, the analysis focused on 19,272 protein-coding genes. Read counts were normalized to counts per million (CPM) to account for library size differences. Genes with low expression levels (CPM < 15 in *in vivo* samples and CPM < 9 in *in vitro* samples) were filtered out to reduce the multiple testing burden and enhance statistical accuracy. Additionally, to account for variability (“wobbling”) in both datasets, the average standard deviation and mean of expression levels across *in vivo* samples for each timepoint were calculated, and genes with values exceeding 0.25 were filtered out. The transcriptome data structure was explored using multidimensional scaling (MDS) *via EdgeR*’s plotMDS() function, where distances approximate typical log_2_ fold changes (FC) between samples. These distances were calculated as the root mean square deviation (Euclidean distance) of log_2_FC values for genes showing significant changes (p-value < 0.05 or Benjamini-Hochberg adjusted p-value = FDR (false discovery rate) < 0.05)) post-vitamin D_3_ supplementation (**Supplementary Tables S2**, **S3**). Mean-Difference (MA) plots were generated using the *DESeq2* package (version 1.42.1). Differential gene expression was analyzed through the *DESeq2* pipeline, which is based on a generalized linear model framework. Gene-wise dispersion estimates were calculated using maximum *a posteriori* estimation, incorporating empirical Bayes shrinkage for improved precision. Normalization was performed using the median-of-ratios method to account for differences in library size. The Wald test was employed to evaluate the significance of differential expression, and p-values were adjusted for multiple testing using the Benjamini-Hochberg procedure.

### 2.5 Analysis of genomic regions for key vitamin D target genes

The genomic regions of key *in vivo* vitamin D target genes were analyzed to identify VDR-binding enhancers and TSS regions using epigenome-wide data from THP-1 cells stimulated for 2 and 24 hours with either 10 nM 1,25(OH)_2_D_3_ or a solvent control (0.1% ethanol). This analysis incorporated ChIP-seq (chromatin immunoprecipitation sequencing) datasets for VDR binding (32). Additionally, FAIRE-seq (26) (formaldehyde-assisted isolation of regulatory elements followed by sequencing) data were utilized to further define regulatory regions *via* chromatin accessibility. The datasets were visualized using the IGV browser (41), highlighting VDR binding enhancer and TSS regions within accessible chromatin, which show responsiveness to 1,25(OH)_2_D_3_ within the TAD regions of vitamin D target genes. A region spanning 0.5 Mb upstream and downstream of each target gene’s TSS was screened; however, only the essential regions are presented in the analysis.

## 3 Results

### 3.1 *In vivo* and *in vitro* transcriptome changes of PBMCs in response to vitamin D

**Figure 1:**
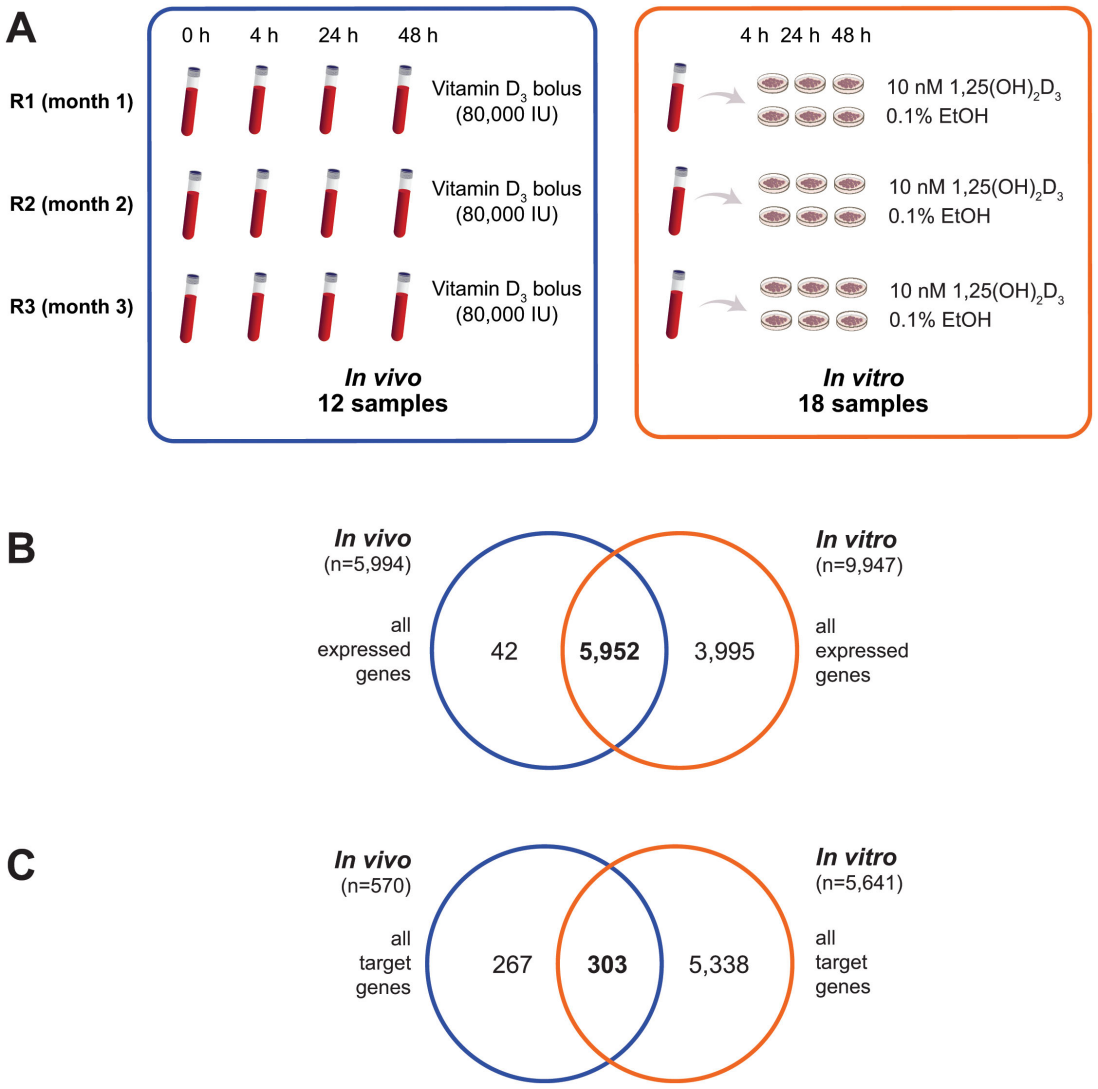
Identification of *in vivo* and *in vitro* vitamin D target genes. The experimental design for identifying vitamin D target genes in both *in vivo* (boxed blue) and *in vitro* (boxed orange) settings is illustrated **(A)**. Venn diagrams are used to depict the overlap between the expressed genes from the *in vivo* study and those from the *in vitro* analysis **(B)**, as well as the overlap between the vitamin D target genes identified *in vivo* and *in vitro* **(C)**.

To compare the transcriptomic response of PBMCs to vitamin D under both *in vivo* and *in vitro* conditions, we utilized the design of the VitDHiD vitamin D intervention trial (37), in which individuals were supplemented with a single bolus of vitamin D_3_ (80,000 IU). In this study, the bolus experiment was repeated over three consecutive months (**Figure 1A**) with one VitDHiD study participant who had been classified as a high responder (42). Blood samples were collected at baseline (prior to supplementation) and at 4, 24, and 48 hours post-supplementation. On average, this resulted in an increase in 25(OH)D_3_ serum levels from 35.5 to 41.9 ng/ml within 48 hours (**Table 1**). Circulating vitamin D_3_ levels rose from 2.6 to 64.8 ng/ml within the first 24 hours and declined to 35.1 ng/ml over the following 24 hours. From each blood sample PBMCs were isolated without further *in vitro* culture (**Figure 1A**, left). In parallel, aliquots of PBMCs collected at baseline (timepoint 0 hours, three biological repeats) had been stimulated *in vitro* with 10 nM 1,25(OH)_2_D_3_ or a solvent control (0.1% EtOH) for 4, 24, and 48 hours (**Figure 1A**, right). The results of the *in vitro* experiment had been published previously (36) and were re-analyzed in the present study using the latest version of the human genome and analysis thresholds standardized across both the *in vivo* and *in vitro* experiments.

**Table 1:** Serum values.

Repeat	Time [hours]	25(OH)D_3_ [ng/ml]	Vitamin D_3_ [ng/ml]
1	0	36.2	3.7
1	4	35.7	26.1
1	24	41.7	65.7
1	48	38.7	34.2
2	0	37.5	2.4
2	4	39.0	20.0
2	24	45.5	73,5
2	48	45.7	38.6
3	0	32.8	1.6
3	4	34.4	16.8
3	24	38.2	55.3
3	48	41.3	32.6

Serum levels of 25(OH)D_3_ and vitamin D_3_ are measured by LC-MS/MS for the three repeats of the time course experiment.

RNA-seq analysis revealed that 5,994 protein-coding genes were commonly expressed in the 12 *in vivo* samples (**Supplementary Table S2**), while 9,947 genes were detected in the 18 *in vitro* samples (**Supplementary Table S3**). Of these, 5,952 genes were shared between both datasets, with 42 genes exclusively expressed *in vivo* and 3,995 uniquely expressed *in vitro* (**Figure 1B**).

**Figure 2:**
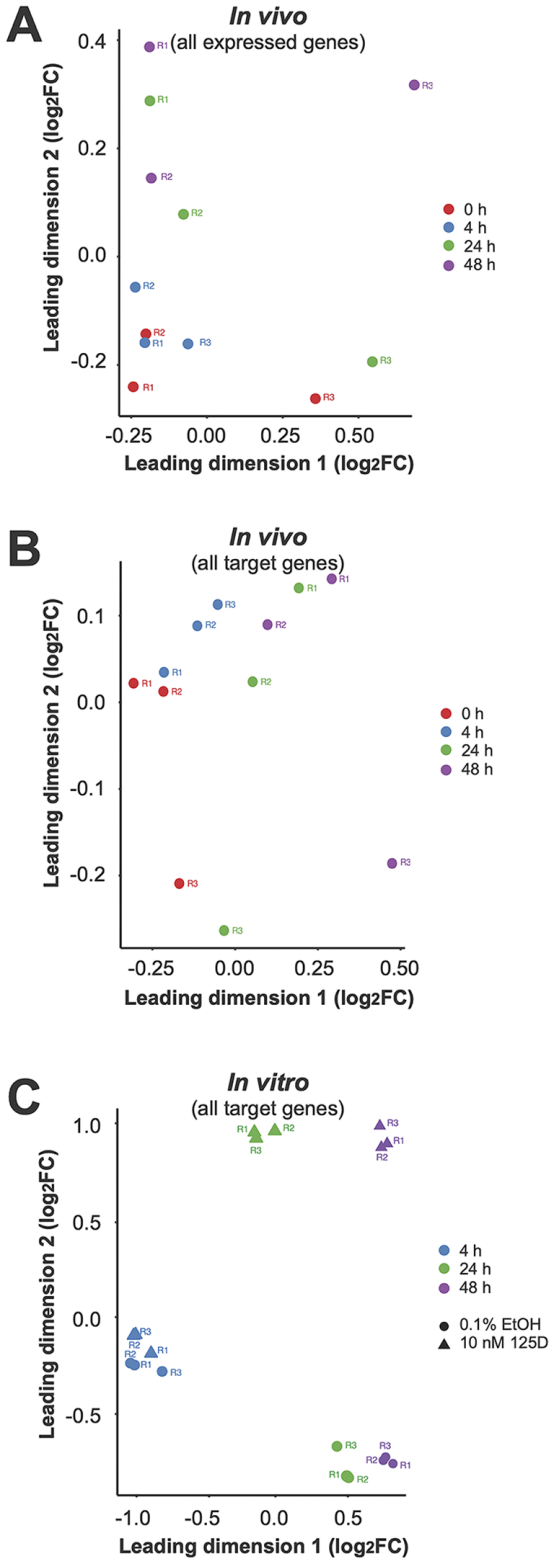
Sample quality assessment *via* MDS. To assess sample quality, MDS was applied to visualize the similarities among the 12 *in vivo* samples **(A, B)** and 18 *in vitro* samples **(C)**. The MDS plots were generated using two different gene sets: all expressed genes **(A, C)** and the 570 *in vivo* target genes **(B)**. These analyses provide insight into the clustering of samples based on overall gene expression patterns and highlight the distinct regulatory effects observed in the identified vitamin D target genes.

Dimensionality reduction using MDS plots highlighted distinct effects of vitamin D. *In vivo*, the second leading dimension showed a clear separation across all three post-supplementation timepoints when analyzing all expressed genes (**Figure 2A**). Notably, when focusing on vitamin D target genes, even the first leading dimension reflected the supplementation effect (**Figure 2B**). In comparison, the *in vitro* samples demonstrated based on all expressed genes a clear distinction between 1,25(OH)_2_D_3_ treatment and solvent control at 24 and 48 hours, but not at 4 hours of stimulation (**Figure 2C**).

Interestingly, differential gene expression analysis identified 570 genes significantly regulated (p < 0.05) by vitamin D_3_ supplementation across at least one of the three *in vivo* timepoints compared to baseline (**Figure 1C**). In contrast, *in vitro* stimulation with 1,25(OH)_2_D_3_ identified a substantial number of 5,641 target genes (p < 0.05), 303 of which overlapped with the *in vivo* target genes. When applying more stringent criteria, such as FDR < 0.05, the number of *in vitro* vitamin D target genes was reduced to 945, with 90 genes overlapping between the *in vivo* and *in vitro* target lists.

In summary, we present a paired study design that allows the parallel investigation of the transcriptomic response of PBMCs from the same individual to vitamin D under both *in vivo* and *in vitro* conditions. Based on p-value significance, 303 protein-coding genes were found to respond significantly to vitamin D within 4–48 hours in both the *in vivo* and *in vitro* settings.

### 3.2 Dynamic response target genes to vitamin D *in vivo* and *in vitro*

**Figure 3:**
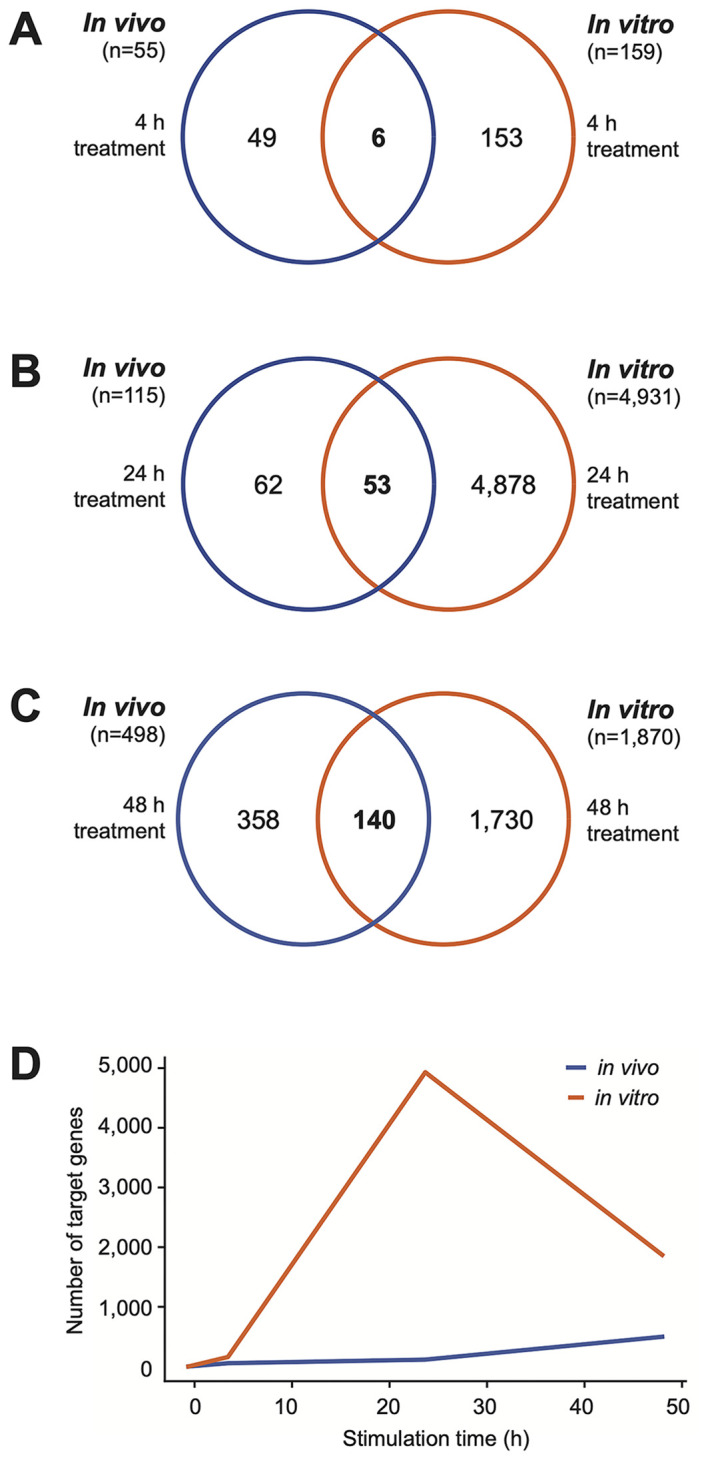
Temporal dynamics of vitamin D target genes. Venn diagrams illustrate the overlap between *in vivo* (blue) and *in vitro* (orange) vitamin D target genes at 4 hours **(A)**, 24 hours **(B)**, and 48 hours **(C)** following the start of the experiments. Additionally, a graph displays the temporal progression in the number of target genes, highlighting the dynamic nature of gene regulation over time in both *in vivo* and *in vitro* contexts **(D)**.

**Figure 4:**
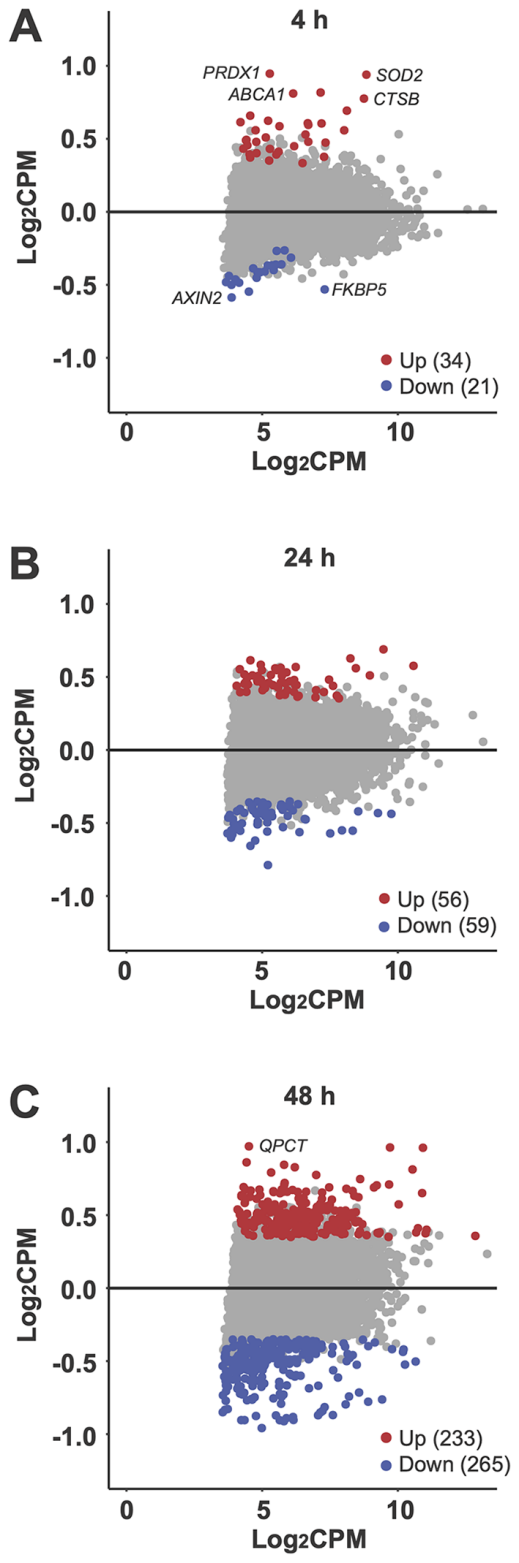
Differential gene expression. MA plots illustrate the impact of vitamin D3 supplementation on all 5,994 commonly expressed genes (CPM > 15) at 4 hours **(A)**, 24 hours **(B)**, and 48 hours **(C)**. Each plot compares the change in expression (log2FC) for each gene with its mean expression level across the compared groups (log2CPM). Genes with significant upregulation (p < 0.05) are highlighted in red, while significantly downregulated genes are shown in blue. Selected genes are labeled for emphasis.

**Figure 5:**
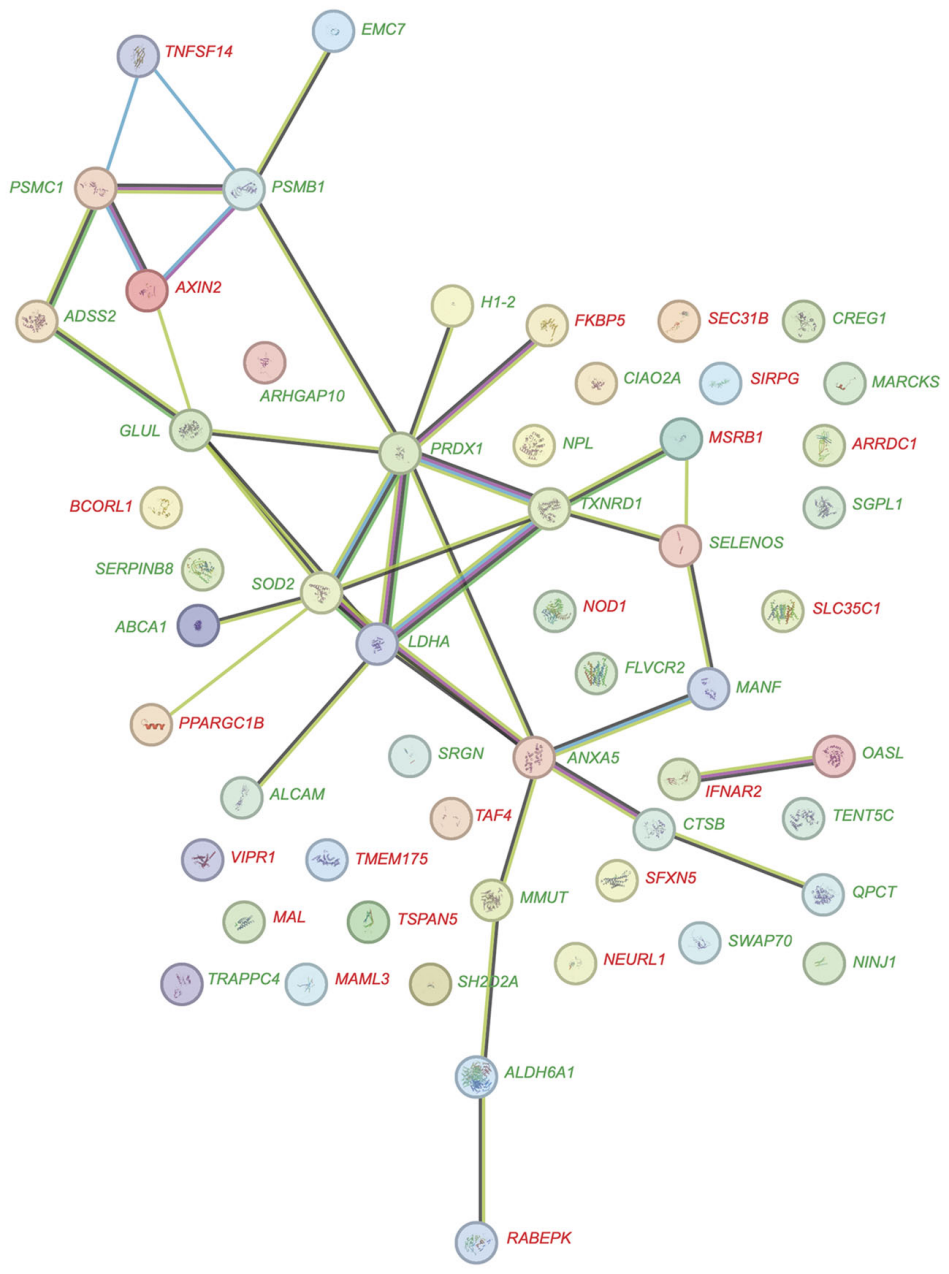
Protein-protein interaction network of *in vivo* vitamin D target genes. The network diagram depicts the interactions among proteins encoded by 55 *in vivo* vitamin D target genes, visualized using the STRING database. Of these genes, 34 are upregulated (green) and 21 are downregulated (red). Each node represents a protein, while edges signify functional and physical associations between them. The thickness of the edges indicates the strength of evidence supporting each interaction, offering insights into the interconnected roles and pathways of these target genes.

A detailed analysis of the dynamic response of PBMCs to vitamin D in the *in vivo* dataset (**Supplementary Table S2**) compared to the *in vitro* dataset (**Supplementary Table S3**) revealed that as early as 4 hours after the start of the experiment, 55 genes were significantly regulated (p < 0.05, absolute log_2_FC > 0.25) by vitamin D_3_ supplementation *in vivo*. In comparison, at this timepoint already 159 genes were regulated by 1,25(OH)_2_D_3_ stimulation *in vitro* (**Figure 3A**). Notably, the two lists of genes share only six in common: *ALCAM* (activated leukocyte cell adhesion molecule), *FLVCR2* (FLVCR choline and putative heme transporter 2), *NINJ1* (ninjurin 1), *PPARGC1B* (PPARG coactivator 1 beta), *SRGN* (serglycin), and *TXNRD1*. Among the 55 early *in vivo* vitamin D-responsive genes, 34 (61.8%) were upregulated and 21 were downregulated (**Figure 4A**). Similarly, among the 159 *in vitro* targets, 103 (64.8%) were upregulated and 56 were downregulated. Analysis using the STRING database (43) revealed that 28 of the proteins encoded by these 55 *in vivo* genes are known to functionally interact (**Figure 5**). Among the overlapping genes, *TXNRD1* appears to play a central role within this interaction network of genes and proteins.

At 24 hours after vitamin D_3_ supplementation, 115 genes exhibit significant changes in expression (p < 0.05, absolute log_2_FC > 0.25), with 56 genes upregulated and 59 downregulated (**Figure 5B**). In contrast, under the same statistical criteria, 1,25(OH)_2_D_3_ regulates the expression of 4,931 genes *in vitro* (**Figure 3B**). Notably, only 53 genes overlap between the two datasets. By 48 hours, the number of genes regulated *in vivo* by vitamin D_3_ supplementation increases to 498, comprising 233 upregulated and 265 downregulated genes (**Figure 4C**). Among these, 140 genes overlap with the 1,870 genes regulated by 1,25(OH)_2_D_3_ *in vitro* (**Figure 3C**).

Taken together, the data indicate a steady increase in the number of *in vivo* target genes over time, rising from 55 at 4 hours to 498 at 48 hours following vitamin D_3_ supplementation. In contrast, the *in vitro* experiments reveal a far greater number of targets, peaking at 24 hours (**Figure 3D**).

### 3.3 Functional profile of early *in vivo* vitamin D target genes

**Figure 6:**
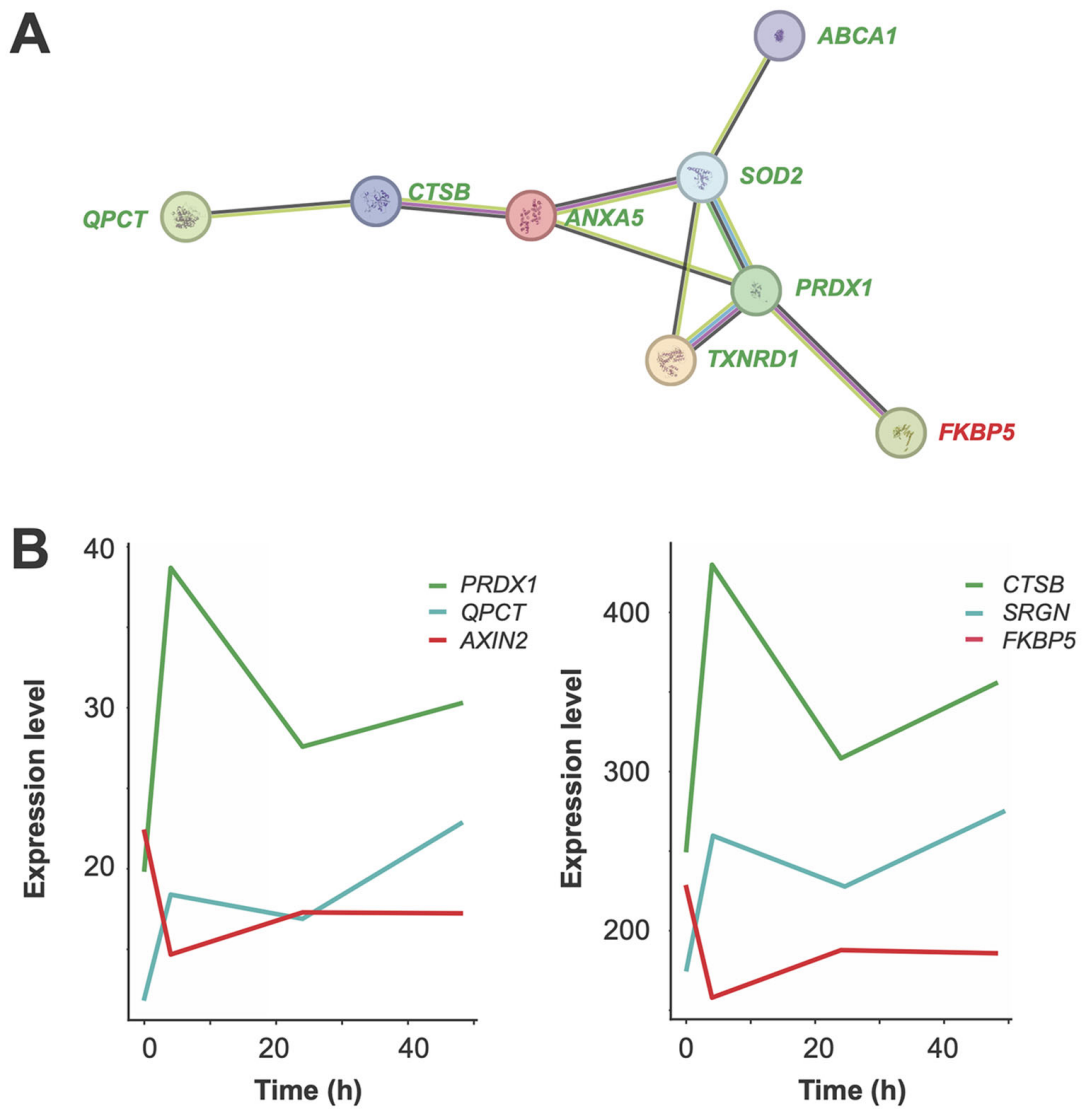
Functional analysis of early *in vivo* vitamin D target genes. A network diagram illustrates interactions among proteins encoded by eight key early *in vivo* vitamin D target genes, visualized using the STRING database **(A)**. Seven of these genes are upregulated (green) and one is downregulated (red). Each node represents a protein, and edges indicate functional or physical associations. The thickness of edges reflects the strength of evidence supporting the interactions, providing insights into the interconnected roles and pathways of these target genes. Graphs depict the expression profiles of representative early *in vivo* vitamin D target genes **(B)**. Genes upregulated with a peak at 4 hours are shown in green, those continuously upregulated in blue, and those downregulated with a peak at 4 hours in red.

Functional profiling of 55 early *in vivo* vitamin D target genes using EnrichR (44, 45) identified the “Selenium Micronutrient Network” from WikiPathways (46) as the top-scoring pathway. This result is primarily due to the inclusion of the early vitamin D target genes *ABCA1* (ATP-binding cassette transporter 1), *MSRB1* (methionine sulfoxide reductase B1), *PRDX1*, *SELENOS* (selenoprotein S), *SOD2*, and *TXNRD1*. Further analysis with STRING, focusing on genes with an absolute log_2_FC change greater than 0.5 at 4 hours post-vitamin D_3_ supplementation, revealed a protein-protein interaction network comprising *ABCA1*, *ANXA5* (annexin A5), *CTSB* (cathepsin B), *FKBP5* (FKBP prolyl isomerase 5), *PRDX1*, *QPCT* (glutaminyl-peptide cyclotransferase), *SOD2* and *TXNRD1* (**Figure 6A**). This network represents a subset of the more extensive protein interaction network depicted in **Figure 5**.

The expression profile of 46 out of the 55 early-responding *in vivo* vitamin D target genes reached maximal up- or downregulation at the 4-hour timepoint (**Supplementary Table S2**). Examples include the upregulated genes *PRDX1* and *CTSB*, as well as the downregulated genes *AXIN2* and *FKBP5* (**Figure 6B**). Additionally, nine genes, such as *QPCT* and *SRGN*, exhibit persistent upregulation.

**Figure 7:**
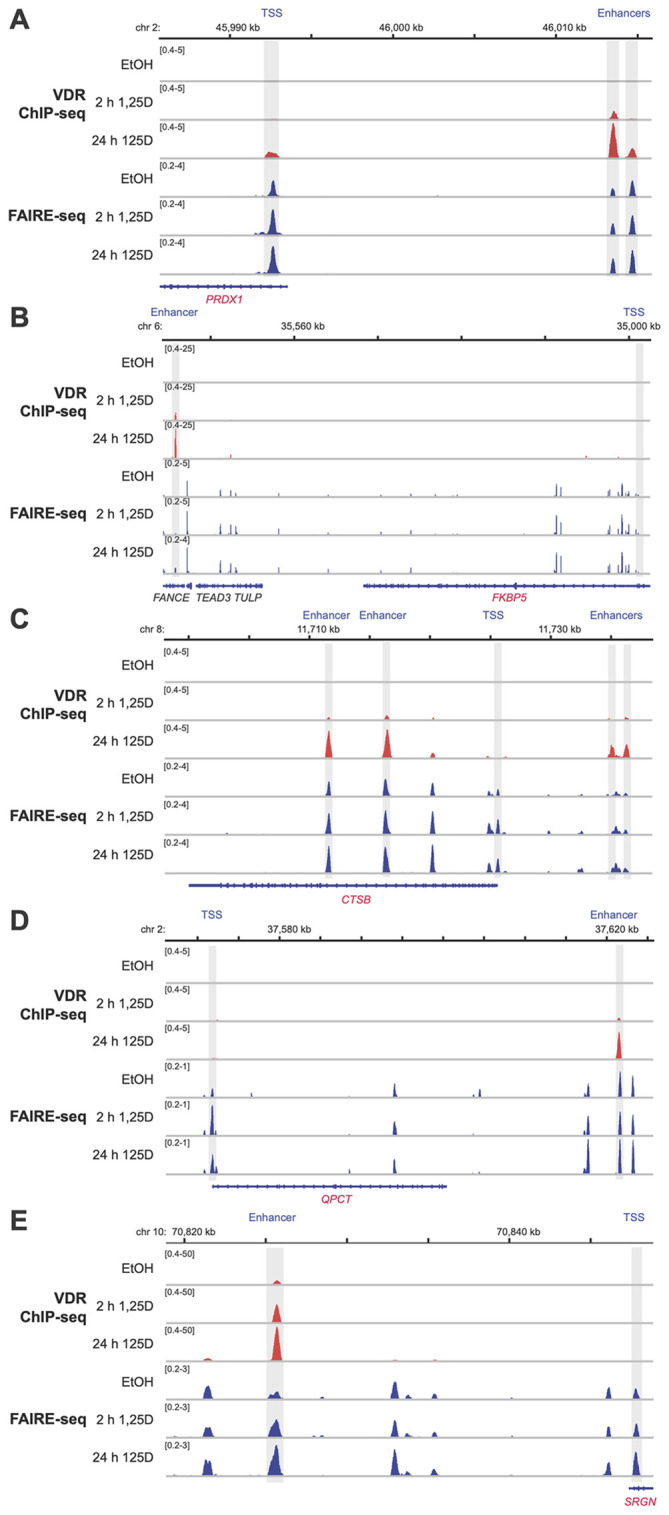
Genomic regions of vitamin D target genes. ChIP-seq results for VDR (red (32)) and FAIRE-seq data (blue (32)) were visualized using the IGV browser, based on experiments conducted in THP-1 cells treated with either solvent (EtOH) or 10 nM 1,25(OH)_2_D_3_ (1,25D) for 2 and 24 hours. The peak tracks represent merged data from three biological replicates. Gene structures are shown in blue, while the vitamin D target genes *PRDX1* **(A)**, *FKBP5* **(B)**, *CTSB* **(C)**, *QPCT* **(D)**, and *SRGN* **(E)** are highlighted in red. Enhancer and TSS regions are shaded in gray. Although genomic regions spanning 0.5 Mb upstream and downstream of each gene’s TSS were examined, only areas relevant to 1,25(OH)_2_D_3_-dependent regulation are displayed.

To investigate potential regulatory mechanisms, the genomic regions within ±500 kb of the TSS of these 55 early target genes were screened for experimentally confirmed VDR binding sites located within accessible chromatin. For this analysis, previously published datasets from VDR ChIP-seq (32) and FAIRE-seq (26) experiments in THP-1 cells, treated with either solvent or 10 nM 1,25(OH)_2_D_3_ for 2 or 24 hours, were utilized. This screening identified at least one VDR-binding enhancer within accessible chromatin for 51 of the 55 genes at a single time point, and for 33 genes at both time points (**Supplementary Table S2**). These enhancers were positioned close enough to the TSS to potentially regulate the corresponding genes. Representative examples include the genes *PRDX1* (**Figure 7A**), *FKBP5* (**Figure 7B**), *CTSB* (**Figure 7C**), *QPCT* (**Figure 7D**), and *SRGN* (**Figure 7E**). Only for the genes *ABCA1*, EMC7 (ER membrane protein complex subunit 7), MAL (mal, T cell differentiation protein), and RABEPK (Rab9 effector protein with kelch motifs) no VDR-binding enhancer has been identified.

In summary, six early vitamin D target genes associated with the selenium micronutrient network emerged as key members of the top-scoring pathways. Among these, four genes (*ABCA1*, *PRDX1*, *SOD2*, and *TXNRD1*) were validated through protein-network analysis. Additionally, for the majority of the 55 early *in vivo* target genes, VDR binding enhancers within accessible chromatin regions were identified, highlighting their potential regulatory mechanisms.

## 4 Discussion

This proof-of-principle study explores the temporal transcriptomic changes in human PBMCs from 4 to 48 hours following a single vitamin D_3_ bolus supplementation. The study design builds on our previous VitDbol (47, 48) and VitDHiD (37, 42) investigations, with key differences: the bolus was administered over three consecutive months, and the first blood samples were collected as early as 4 hours post-supplementation. The triple biological replicate design of this *in vivo* experiment facilitates robust statistical analysis of gene expression changes at the individual level. This contrasts with the cohort design of the VitDbol and VitDHiD studies, in which each participant received a single vitamin D_3_ bolus, and statistical analysis was enabled through the inclusion of multiple individuals. Importantly, the inclusion of the 4-hour time point allows for differentiation between primary and secondary vitamin D target genes. Additionally, *in vitro* stimulation of PBMCs from the same individual with the biologically active form of vitamin D_3_, 1,25(OH)_2_D_3_, serves as a critical reference point, bridging findings from this study to the extensive body of cell culture research that has dominated vitamin D-related gene regulation studies over the past three decades (9, 10, 49, 50).

Across all three timepoints, we identified a total of 570 *in vivo* vitamin D target genes, of which the majority (53.2%) were also detected through *in vitro* stimulation with 1,25(OH)_2_D_3_ of PBMCs of the same individual. As 1,25(OH)_2_D_3_ is a nuclear hormone and classified as a medical compound, direct intervention studies using this vitamin D metabolite in healthy individuals are not allowed. Moreover, it is ethically prohibited to induce severe vitamin D deficiency in humans by prolonged deprivation. Therefore, *in vivo* vitamin D studies in healthy individuals can only be conducted within the range of vitamin D sufficiency to avoid potential harm to participants. Vitamin D_3_ is rapidly absorbed in the intestine, as evidenced by an increase in serum levels from 2.6 to 21.0 ng/ml within 4 hours post-supplementation. It is subsequently converted in the liver and kidneys into 25(OH)D_3_ and 1,25(OH)_2_D_3_, respectively (51). This is reflected by a 6.3 ng/ml rise in 25(OH)D_3_ levels within 24 hours. Notably, in the context of our VitDbol study, we demonstrated that an oral vitamin D_3_ bolus also significantly elevates serum 1,25(OH)_2_D_3_ levels, thereby validating the use of its precursor, vitamin D_3_, for investigating downstream gene regulatory effects *in vivo* (47). Basal *in vivo* serum levels of 1,25(OH)_2_D_3_ are approximately 0.1 nM, and a vitamin D_3_ bolus increases these levels by 20–30% (47). In contrast, *in vitro* experiments typically involve stimulations with 10 nM 1,25(OH)_2_D_3_ compared to a vitamin D-free solvent control (26). Furthermore, *in vitro* studies are conducted under highly controlled and standardized conditions with minimal external interference, whereas *in vivo* setups are subject to significant environmental and lifestyle variability. These factors contribute to the observed differences: despite using identical statistical thresholds and restrictions, we identified significantly more vitamin D target genes *in vitro* across all three timepoints than under *in vivo* conditions. Moreover, genes commonly used as *in vitro* markers, are either not expressed like in the case of *CAMP* (cathelicidin antimicrobial peptide) (52) due to the absence of bacterial infection in the study participant, or not significantly induced, as with *CYP24A1* (cytochrome P450 family 24 subfamily A member 1) (10), which may reflect its higher basal expression *in vivo* and the comparatively modest increase in 1,25(OH)_2_D_3_ levels. This discrepancy emphasizes the challenges inherent in translating *in vitro* findings to the more complex *in vivo* environment.

Since the 24- and 48-hour time points have already been extensively studied in the context of identifying *in vivo* vitamin D target genes, our work focuses on early responding genes (42, 47, 48). We identified 55 genes that showed a significant response as early as 4 hours after vitamin D_3_ supplementation *in vivo*. Notably, half of these genes encode a protein network, with its core components linked to the micronutrient selenium. EnrichR analysis suggests that *SELENOS* is among the key target genes that elucidate the functional role of vitamin D in the early hours following supplementation. The SELENOS protein is essential for maintaining endoplasmic reticulum function by promoting the removal of misfolded proteins, thereby playing a critical role in key processes such as inflammation regulation and antioxidative defense (53). Vitamin D has established roles in modulating antioxidant pathways, including those involving glutathione metabolism (54). Our findings suggest that vitamin D enhances SELENOS-mediated processes, offering cellular protection against damage from free radicals. Interestingly, both vitamin D deficiency and SELENOS dysfunction have been linked to chronic conditions such as type 2 diabetes, cardiovascular diseases, and neurodegenerative disorders (55). This overlap highlights the possibility of a collaborative influence of vitamin D and SELENOS on metabolic pathways essential for preventing these diseases.

A primary vitamin D target gene is defined by the presence of an enhancer with a VDR binding site within the same TAD. This criterion is met by 51 of the 55 genes that respond within just 4 hours of vitamin D_3_ supplementation. In contrast, a secondary vitamin D target gene does not require a VDR-containing enhancer for its regulation. Instead, its transcriptional activity is modulated by transcription factors, cofactors, or chromatin modifiers encoded by primary vitamin D target genes. Notably, among the identified primary *in vivo* vitamin D target genes, only a few, such as *BCORL1* (BCL6 corepressor-like 1), *CREG1* (cellular repressor of E1A-stimulated genes 1), *MAML3* (mastermind-like transcriptional coactivator 3), *PPARGC1A*, and *TAF4* (TATA-box binding protein-associated factor 4), appear to have the capacity to mediate the secondary effects of vitamin D.

The primary *in vivo* vitamin D target genes *PRDX1*, *SOD2*, and *TXNRD1* encode enzymes that play essential roles in the cellular antioxidant defense system, primarily by managing oxidative stress and maintaining redox homeostasis (56). PRDX1 functions as a peroxidase, reducing hydrogen peroxide and organic hydroperoxides to water and alcohol, respectively. This activity mitigates oxidative damage and protects cellular components from reactive oxygen species (57). SOD2 catalyzes the dismutation of superoxide radicals into hydrogen peroxide and oxygen, serving as a critical defense against oxidative damage caused by superoxide radicals generated during mitochondrial respiration. TXNRD1 is responsible for regenerating the reduced form of thioredoxin, which subsequently reduces disulfide bonds and scavenges reactive oxygen species, thus contributing to cellular redox balance. By upregulating the genes *PRDX1*, SOD2, and *TXNRD1*, vitamin D enhances the cell’s ability to combat oxidative stress, reinforcing a robust antioxidant defense system critical for cellular integrity and function.

The present conclusion that vitamin D modulates the redox response of immune cells aligns with a growing body of evidence linking vitamin D to cellular defense mechanisms against various forms of stress and intoxication, including damage from prolonged UV exposure. For instance, vitamin D_3_ and its hydroxy-derivatives produced *via* the CYP11A1 pathway have demonstrated significant protective effects against UVB-induced cellular injury, primarily through activation of the redox-sensitive transcription factor NFE2L2 (NFE2 like BZIP transcription factor 2, also known as NRF2) (58). NFE2L2 plays a central role in orchestrating the antioxidant defense system by regulating the expression of key detoxifying and antioxidant enzymes such as glutathione reductase, heme oxygenase 1, catalase, and SODs. While these protective mechanisms have been well-documented in human keratinocytes, their relevance to immune cells, particularly PBMCs, remains to be validated. Notably, in the time frame analyzed in this study (0–48 hours), vitamin D_3_ supplementation did not result in significant changes in NFE2L2 expression in immune cells. Based on a meta-analysis of transcriptomic datasets, a core set of 14 target genes of NFE2L2 has been identified (59), but none of them overlap with the 570 high-confidence *in vivo* vitamin D target genes reported in this study. In contrast, 10 of the 14 genes are found among the 5,461 *in vitro* targets of 1,25(OH)_2_D_3_. Nevertheless, a hypergeometric test revealed that this overlap is not statistically significant (p = 0.201). While the potential crosstalk between vitamin D and NFE2L2 signaling remains an interesting hypothesis, our findings do not provide statistically significant evidence to support a functional connection.

Based on a meta-analysis of transcriptome datasets of NFE2L2 targets a core list of 14 genes have been identified (59). However, none of these 14 belongs to the 570 *in vivo* vitamin D target. In contrast, 10 of the genes are contain in the list of 5,461 *in vitro* targets of 1,25(OH)_2_D_3_. However, a hypergeometric test indicates that this finding is not significant (p=0.201). Although this topic should be further explored, our study does not provide any significant evidence for a functional inferences between the signaling of vitamin D and NFE2L2.

This study has several limitations. First, this proof-of-principle trial was conducted with a single individual and should therefore be considered a case study, from which only limited conclusions can be drawn for the general population. Consequently, the findings need to be validated in a larger cohort. Second, in order to investigate the effects of vitamin D on PBMCs under human *in vivo* conditions, we limited the handling of PBMCs and avoided additional *in vitro* stressors, such as monocyte isolation or single-cell isolation methods (60). Third, the study focuses on the transcriptome, which serves only as a partial proxy for protein levels (61), limits the interpretation of functional outcomes. Therefore, the findings and their functional implications require validation through proteome-wide data and functional assays. Finally, a denser sampling schedule at earlier time points would provide a more precise distinction between primary and secondary targets.

In conclusion, this study uniquely identifies a set of 55 early *in vivo* vitamin D target genes in human PBMCs, with 51 of them confirmed as primary targets through the presence of VDR-binding enhancers. Notable members of this gene set include *SELENOS*, *PRDX1*, *SOD2*, and *TXNRD1*, which encode proteins central to antioxidant defense. These findings establish a connection to detoxification, one of the most ancient evolutionary functions of vitamin D (5, 62) having a central role in modulating innate immunity.

## Data Availability

The datasets presented in this study can be found in online repositories. The names of the repository/repositories and accession number(s) can be found in the article/**Supplementary Material**.
